# Time Series of Potential US Wildland Fire Smoke Exposures

**DOI:** 10.3389/fpubh.2020.00126

**Published:** 2020-04-21

**Authors:** Jason A. Vargo

**Affiliations:** California Department of Public Health, Climate Change and Health Equity Program, Richmond, CA, United States

**Keywords:** exposure, air pollutants, climate change, wildfire, smoke, remote sensing

## Introduction

As the threats of climate change become more immediate and persistent, there is a growing need for datasets to document the burden of climate-related events and exposures on human health over time. These data should be freely available, timely and long-running, spatially resolved, and consistent. This data report presents a new dataset for understanding the potential burden of smoke related to wildland fires (wildfires) on communities across the United States since 2010. The dataset combines data from the National Oceanic and Atmospheric Administration's (NOAA) Office of Satellite and Product Operations Hazard Mapping System's Smoke Product (HMS Smoke) with United States Census Block Group Centers of Population to estimate potential exposures to light, medium, and heavy categories of wildfire smoke. The result is a daily assignment of each of the 220,334 2010 U.S Block Groups for each of the HMS Smoke categories and includes 2010 Census population counts. This database can be used to identify populations potentially exposed to wildfire smoke on a given day or to calculate the potential person-days of wildfire smoke exposure for a specified period or spatial unit of interest. Using state, county or tract identifiers included in the database, aggregation to these familiar units of the US Census topology can be accomplished without the use of a geographic information system (GIS). This data report describes the datasets combined to produce this potential wildfire exposure database and outlines some basic use cases and ideas for future work.

It is fundamental to understand the methods, strengths, and limitations of the underlying information used to create this dataset. Most important is the HMS Smoke Product from NOAA's Office of Satellite and Product Operations (OSPO). HMS uses visible imagery from satellites to generate smoke plumes associated with fires. Although there are many air quality datasets available, HMS is particularly valuable as it is specific to fires, which are automatically detected using several satellites and algorithms (1–4). Trained analysts within OSPO modify fire detections to improve accuracy and examine visible imagery from two Geostationary Operational Environmental Satellites (GOES) to identity smoke plumes associated with detected fires. GOES-10 and GOES-12 collect infrared information every 15 min, but with lower spatial resolution (4 km from the 3.9 μm band) than polar orbiting instruments. Visible imagery is available at 1 km spatial resolution. Polar orbiting instruments including the Moderate Resolution Imaging Spectroradiometer (MODIS) (both AQUA and TERRA) and the Advanced Very High Resolution Radiometer (AVHRR) collect imagery less often than geostationary satellites (e.g., GOES) but make use of passes that take advantage of favorable viewing angles for smoke detection (low angle of solar incidence). Over much of North America (low and mid-latitudes) these passes occur in the morning, just after sunrise and in the evening just before sunset (5). Aerosol Optical Depth information collected from GOES satellites, the GOES Aerosol and Smoke Product (GASP) (6), are used to provide an objective and quantitative estimate of smoke density. A smoke density is then assigned to each plume using the aerosol optical depth (AOD) information which accompanies each GOES pixel. Plumes are categorized as light, medium, or heavy and categories approximately correspond to fine particulate matter (PM_2.5_) concentrations of 0–10, 10–21, and 22+ μg/m^3^, respectively. The HMS Smoke product for each day over North America are released ~72 h after collection.

There are factors of the HMS Smoke data which affect its precision as a standalone estimator for wildland fire smoke exposure. The concentrations associated with each HMS Smoke density category are not exact and do not necessarily correspond to ground-level concentrations. Also, differences in the spatial resolution of GASP AOD information and the visible band of GOES may understate the concentrations of smaller smoke plumes. A second drawback of using the visible imagery is that it is affected by cloud cover and unable to differentiate elevations or to determine the height of a plume. The HMS Smoke product is also generated completely from satellite passes occurring during daylight hours. There is no nighttime observation of wildfire smoke in the database; however, the data integration of multiple instruments into the HMS Smoke products allows for the collective strengths to overcome individual limitations (5).

Despite these limitations, HMS has been validated and shown to correlate with elevated PM_2.5_ concentrations at ground-level monitors. A 2015 study examined two large fire events and 13 AQS monitors near California's Central Valley and Western Sierra Nevada. Heavy HMS Smoke plumes were found to correspond to an exceedance of the station's 96th percentile value more than one third of the time (36%) and were shown to increase the daily 96th percentile of stations by 14 μg/m^3^. Medium HMS Smoke plumes also corresponded to exceedences of the 96th percentile, but less frequently. When combined with statistical techniques to consider weather and seasonal factors, the study showed that HMS Smoke plumes could reliably identify periods of wildfire influence in the AQS record with 95% accuracy (7). A 2018 study used HMS and AQS data from 2006 to 2013 to estimate the effect of plums on ozone and PM_2.5_ measurements using generalized additive models. The results showed a disproportionate number of days considered “unhealthy” by USEPA's Air Quality Index (AQI) occurring on days when HMS Smoke plumes are present. Unhealthy days were 3.3 (O_3_) and 2.5 (PM_2.5_) times more likely to occur when HMS Smoke Plumes were present than when absent (8). Health impacts of exposure to poor air quality have also been demonstrated using HMS Smoke information. In 2017 a study examining California's 2015 wildfire season found that risk of heart attack and stroke increased (22 and 18%, respectively) 1 day following the presence of HMS Smoke plumes over an individual's county of residence. The risk of heart attack and stroke, particularly for adults over the age of 65 increased by 42 and 22%, respectively 1 day after heavy smoke plumes in the county and risk increased with plume density (9).

This data report presents a modest advancement of NOAA's HMS work with the aims of spurring additional work on the impacts of wildfire smoke on the health of US Populations. Namely, these should include tracking potential wildfire smoke exposures to identify areas and times most heavily impacted by smoke, adding potential smoke exposures to population characteristics describing the social determinants of health in order to better distribute resources and contextualize public health messages and interventions, and combining information specific to wildfire smoke with other air pollution data to better isolate and understand the contribution of wildfires to poor health.

## Methods

**Figure 1:**
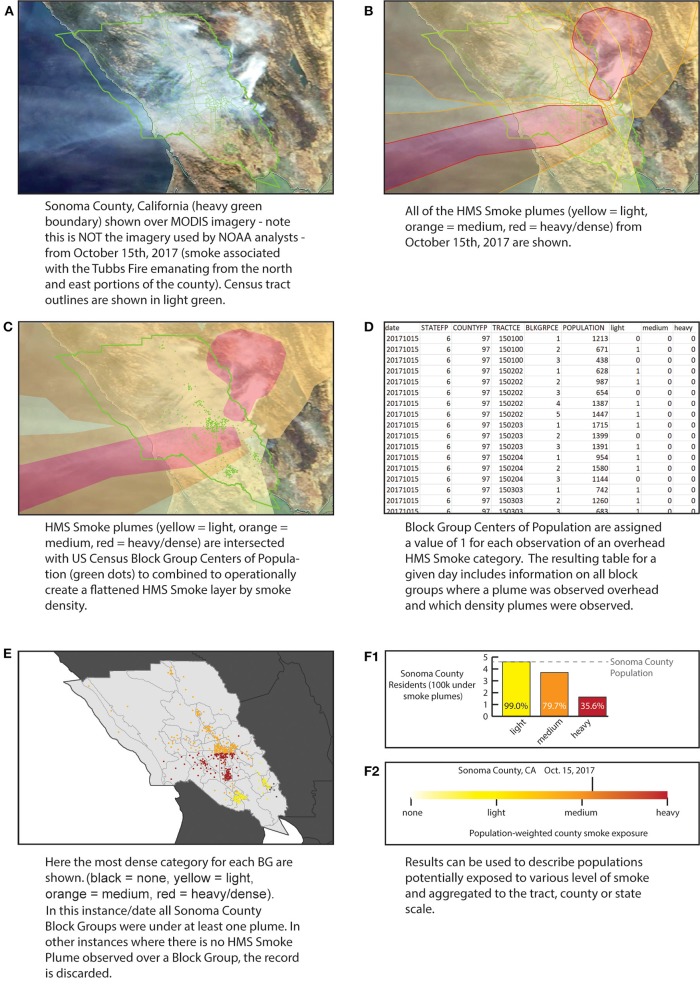
Processing of the data using example of Sonoma County Oct. 11, 2017. **(A)** Example visible imagery; **(B)** HMS Smoke plumes layer for Oct. 11, 2017; **(C)** Simplification of all plumes by density; **(D)** resulting table of the intersection of plumes and centers of population; **(E)** Resulting maximum density smoke assignment for Sonoma Block Group centers of population; **(F1)** Total number of Sonoma residents under each density category; **(F2)** Population-weighted smoke density for Sonoma County.

HMS Smoke data were obtained from NOAA's Online archive of the HMS data, available at https://satepsanone.nesdis.noaa.gov/pub/volcano/FIRE/HMS_ARCHIVE/. Though the archive extends back to 2003, only years 2010–2019 are employed. HMS Smoke began including density information in 2007 and earlier records exist which describe smoke presence using a text format, though without density information. Due to gaps in the density information in the archival record in 2008, 2009, the dataset here is limited to June 2010 and beyond. This was done in order to have a resulting time series with consistent smoke density information. HMS Smoke layers for a specific day are created from several satellite passes (example in Figure 1A), and so multiple plumes may exist over any single location on a given day (Figure 1B). To resolve plumes to one observation for each day and location, a single day's plumes are treated as flattened layers so that the coverage of smoke plumes of specific densities is defined by the appearance of a plume of that density from any HMS collection in that day (Figure 1C).

Information on population was obtained from the 2010 US Census Centers of Population. The entire set of 2010 Block Group Centers of Population is available as a single text file at https://www2.census.gov/geo/docs/reference/cenpop2010/blkgrp/CenPop2010_Mean_BG.txt. The latitude and longitude fields from the Centers of Population file were used to create a spatial file of points and intersected with HMS Smoke plumes. The concept of the center of population as used by the US Census Bureau is that of a balance point. Using the Block Group Centers of Population, rather than geographic centroids or cadastral boundaries helps reduce exposure misclassification, particularly in rural or more sparsely populated areas. The block group scale is the finest scale for which the Centers of Population exist and they were used to best represent the locations where populations within Census units reside. The use of points also simplified the combination of plumes with populations spread out over land.

To combine HMS Smoke plume information with US populations, a function written in R and implemented with RStudio was employed. For each day, the function downloads, and unzips as necessary, the appropriate GIS shapefile of that day's HMS Smoke plumes. Once unzipped to temporary memory, these files are intersected with the US Centers of Population points, though the full script can be modified to intersect plumes with other points of interest (for example, AQS monitors). The full script for processing can be accessed and amended at https://github.com/vargovargo/WFSmokeExp/blob/master/processHMS.R.

The results of this spatial intersection for each day are output as a comma separated values (csv) with the date in the file name. The result is a table of locations and smoke observations for a given day (Figure 1D). Each row in the table is a record of a single block group and day combination. Data is stored as csv with columns (names in bold):

- The date of the observation as **“date”** formatted as “yyyymmdd” (string).
- The state identifier for the block group as **“STATEFP”** (integer).
- The county identifier for the block group as **“COUNTYFP”** (integer).
- The tract identifier for the block group as **“TRACTCE”** (integer).
- The block group identifier for the block group as **“BLKGRPCE”** (integer).
- The population of the block group in the 2010 US Census as **“POPULATION”** (integer).
- An indicator of whether that block group fell under an HMS Smoke plume of “light” density smoke on that day as **“light”** (integer 0 or 1).
- An indicator of whether that block group fell under an HMS Smoke plume of “medium” density smoke on that day as **“medium”** (integer 0 or 1).
- An indicator of whether that block group fell under an HMS Smoke plume of “heavy” or “dense” smoke on that day as **“heavy”** (integer 0 or 1).

Only those locations with any plume overhead are included in the final table for that day. This is done to reduce the file size of each day's smoke-location result. Multiple days can easily be combined to produce a full US dataset. This combined dataset is what is available through a public repository available through Harvard's Dataverse at https://dataverse.harvard.edu/dataset.xhtml?persistentId=doi:10.7910/DVN/CTWGWE. The resulting table can be subset to isolate periods and locations of interest. These can be connected to geographic location information and mapped (Figure 1E), or aggregated to larger census-relevant boundaries for further analysis (example in **Figures 1F1,F2**). In the next section, some examples of such operations are provided.

## Ways To Interpret and Reuse the Data

The current dataset of potential wildland fire smoke exposures covers June 1, 2010 through December 17, 2019 and has 59,301,641 records. The dataset can be expanded to all day-location combinations (including when smoke is not present); however, to avoid a file size above 2GB, that is not provided. It is important to note that the dataset represents a first-pass description of potential exposures to wildfire smoke, which can and should be investigated more closely and in connection with complementary datasets to more precisely understand any individual exposure.

**Figure 2:**
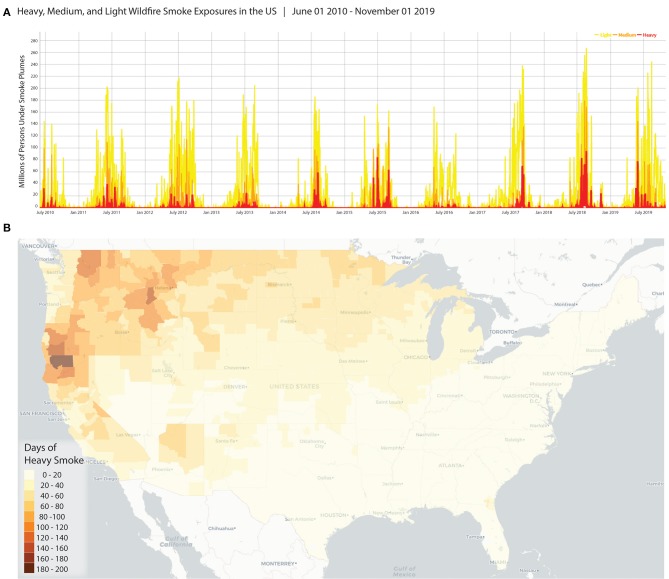
Examples of how the data may be visualized and reused. **(A)** Shows a time series for the number of people under different categories of HMS Smoke for the full dataset (all US, June 2010–Nov 1, 2019); **(B)** The number of days of heavy smoke for any part of the county (contiguous US, June 2010–Nov 1, 2019).

On a population basis, these data do a fair job of tracking exposures for a specific geography over a period. Figure 2A is produced using the full smoke dataset and sums the product of (a) the smoke indicator (0,1) and (b) the population by day for all the block groups within the area of interest. These operations are easily performed for states or counties without requiring a Geographic Information System (GIS). The data and resulting plots can be used to describe trends in wildland fire smoke season durations (start and end dates) and periods of greatest and or longest exposures. Understanding trends is important for studying prolonged and chronic exposures to wildland fire smoke. This can be helpful for studies seeking to document burden, examine chronic exposure, and quantify impacts of air pollution related to smoke, specifically. Also, understanding seasonality, especially for smoke which may impact populations impacted far from fires, can be useful in guiding investments and messages for preparation and protection.

A “person-days” metric is one way to present the results from this dataset and provides a useful way of tallying potential exposure, particularly for large areas with widely varying population densities. The use of person-days under different smoke plumes has been used in research to describe exposures previously (10, 11). Presentation of results as person-days may emphasize the burden of wildland fire smoke on densely populated areas and understate the more frequent exposures occurring in rural areas.

The dataset can also be used to compare locations during a specific time. The map in Figure 2B shows the number of days of heavy smoke overhead by county (Contiguous US) for the period June 1, 2010—November 1, 2019. In this case, the maximum value for each smoke density category is assigned to the larger spatial unit, so that if any block group in the county was under heavy HMS Smoke for a day the county is considered to experience that day with potential heavy smoke exposure. Alternatively, a population-weighted smoke level can be calculated for counties by treating the smoke levels as numerical values (i.e., none as 0, light as 1, medium as 2, heavy as 3) and weighting the maximum smoke value for each block group according to the proportion of the county population (see Figure 1F2). In both cases, a GIS is required only for presentation; the creation of the data can be achieved without spatial operations. These data can be important for targeting funding, interventions, and communications to areas more often impacted by smoke from fires. Similarly, finer scale investigations can shed light on specific parts of the county most heavily impacted, as shown in the Sonoma County Example (Figure 1E). Such maps and information can help with after-incident reports and preparation for future fire and smoke events. They may assist authorities to improve the siting of evacuation centers and routes.

These data can also be useful for health research focused on wildland fire smoke to identify periods and locations of potential exposure and sampling for studies. For example, researchers looking for new biomarkers of wildfire smoke may wish to identify a cohort of potentially exposed and unexposed participants from which to obtain samples.

Moving forward the methods used to produce this dataset can be modified for other data on empirical air quality measurement. Demonstrations by previous analyses linking HMS Smoke plumes and elevated PM_2.5_ measurements (7) can be expanded or paired with newer sources of air quality data, including combinations of remotely sensed, monitored, and modeled information. New methods and processing are increasingly being explored to improve the precision and resolution of smoke-related air pollution (12, 13). Clearer identification of wildfire's influence on air pollution will help scientists, forest managers, and decision makers to understand the impact of different types of burns on air quality. The impact of prescribed burns on local air quality is of particular importance for local forest management decisions. Understanding the relative impacts of different management practices and the frequency of large, full suppression fire events is required to justify more proactive and preventative fire management practices.

This dataset should also be used to understand the populations most affected by wildfire smoke through combination with population characteristics which describe inequitable vulnerability. The use of US Census units in the data (block groups) allows for population characteristics to be easily combined with smoke exposures to identify disproportionate or inequitable burden. For example, combining the dataset with characteristics including age, race, poverty, outdoor occupations, and others from the US Census can help to highlight more sensitive, less well-resourced, or vulnerable populations for targeting intervention and protection.

There remains room for improvement with understanding exposures to wildland fire and smoke; however the need for tools and data to inform actions to protect human health is immediate. This dataset begins to address that need for an environmental health exposure that is increasing as the climate emergency continues. By providing a means to track and describe the potential wildfire smoke exposures over time in a consistent manner, this dataset can improve our understanding of populations particularly affected by smoke, guide exposure reduction, and assess longer-term land management strategies.

## Data Availability Statement

The dataset described in this report can be found on the Harvard Dataverse, available at https://dataverse.harvard.edu/dataset.xhtml?persistentId=doi:10.7910/DVN/CTWGWE.

## Author Contributions

JV contributed to this work from conception through completion, including manuscript composition, figure creation, and data processing.

## Conflict of Interest

The author declares that the research was conducted in the absence of any commercial or financial relationships that could be construed as a potential conflict of interest.
